# ACCESSING LONG-TERM SERVICES AND SUPPORTS: HOW DO ETHNIC MINORITIES NAVIGATE THE SYSTEM?

**DOI:** 10.1093/geroni/igz038.711

**Published:** 2019-11-08

**Authors:** Lauren Ring, Allen Glicksman

**Affiliations:** Philadelphia Corporation for Aging, Philadelphia, Pennsylvania, United States

## Abstract

The decision to seek Long Term Services and Supports (LTSS) can be challenging for older adults and family members. These challenges can be greater for members of certain ethnic/cultural minority communities who are not fluent in English. Our study examines the ways in which older adults in limited English-speaking communities (Spanish / Mandarin Chinese) navigate the use of LTSS. The findings will be used to evaluate disparities in service perception and access experienced by these populations. Our research examines the ways in which information is shared among community members and how they identify trusted sources of information. Ultimately, we wish to examine how these social networks and trusted neighborhood institutions do, or do not, connect older adults in need to the formal LTSS system. We use a modeling technique called Social Interaction Modeling (SIM), which allows for the inclusion of both conceptual and data based elements, to frame this process.

